# A Systematic Review and Meta-Analysis of Human Leukocyte Antigen-DR (HLA-DR) in Onychomycosis: HLA-DR8 Confers Susceptibility

**DOI:** 10.7759/cureus.69162

**Published:** 2024-09-11

**Authors:** Andrew C Cook, Nathan E Cohen, Rishi Patel, Shannon South, Marcia C Ballantyne

**Affiliations:** 1 College of Osteopathic Medicine, Lake Erie College of Osteopathic Medicine - Bradenton, Bradenton, USA; 2 Pathology, Lake Erie College of Osteopathic Medicine - Bradenton, Bradenton, USA

**Keywords:** allele frequency, fungal nail infection, genetic association, hla-dr, human leukocyte antigen (hla), meta-analysis, onychomycosis, prisma guidelines, systematic literature review, trichophyton rubrum

## Abstract

Onychomycosis (OM) is a nail infection from various fungal species, representing a worldwide dermatologic health concern. The toenails are most often affected. Comorbid chronic health conditions and environmental and genetic factors play a role in the development of OM. It has been observed that certain populations have an increased risk of developing OM, suggesting an inherited component to its etiology. Recent studies have observed the impact of the human leukocyte antigen-DR (HLA-DR) profile on the likelihood of developing OM; however, none have aggregated these studies for a meta-analysis to determine a statistical effect. The literature was systematically reviewed following Preferred Reporting Items for Systematic Reviews and Meta-Analyses guidelines to determine the effect of the HLA-DR profile on OM susceptibility. Studies that contained HLA-DR allele frequency data on patients with OM were included. Studies that contained too much allele frequency data, did not contain HLA-DR allele frequency data, or were written in a non-English language were excluded. Google Scholar, PubMed, and Scientific Direct databases were searched. The risk of bias was assessed by using the National Institutes of Health (NIH) quality assessment case-control study tool. The results were generated using Review Manager version 5.4 by extracting and inputting HLA-DR allele frequency data into the program. The program created aggregated odds ratios that were visually represented in forest plots. A total of five articles were included in the analysis. One hundred fifty-six patients with OM were used in this analysis. Mexican mestizos and United States Caucasian populations were represented in this study. Overall, the NIH risk of bias tool revealed that most studies included did not justify their sample size, or the assessors were not blinded. Of all the HLA-DR alleles analyzed, only HLA-DR8 revealed a statistically significant result with an odds ratio of 1.70 with a 95% CI (1.05-2.76). This suggests that HLA-DR8 confers a 70% higher risk of susceptibility to OM. This finding can help identify these target populations and serve as the basis for personalized treatment solutions.

## Introduction and background

Onychomycosis (OM) represents about half of most toenail health concerns of patients worldwide, with a prevalence of 5.5% [1]. Dermatophytes (DP), yeasts, and non-DP molds have been implicated as causal agents. Among the DPs, *Trichophyton rubrum* is the most prevalent, responsible for 90% of cases in North America [2]. Outside of this region, certain OM agents establish certain niches. For example, in warmer climates, non-DP molds such as* Aspergillus flavus*, *Scopulariopsis brevicaulis*,* Fusarium* species, *Scytalidium dimidiatum*, and *Acremonium *species are more prevalent [3]. *Candida* species infections are frequently seen in patients with digit exposure to water [1]. OM can be due to a variety of environmental, comorbid, or genetic etiologies. Although the environmental and comorbid causes are widely known, recent research interest has investigated the roles genetics play in susceptibility.

The two major underlying comorbid risk factors typically involve either circulation deficits or an abnormal immune response. The major cause of altered circulation is found in patients with peripheral arterial disease, often due to smoking. Patients who have a human immunodeficiency virus (HIV) infection with a cluster of differentiation (CD) 4+ count of 370 cells/mm^3^ are associated with an increased risk of OM [4]. Diabetes mellitus causes both microvascular and an altered polymorphonuclear neutrophil response associated with abnormal carbohydrate regulation, leading to increased* Candida albicans* OM [5-6]. Patients with inflammatory bowel disease are more susceptible to DP infection due to inadequate cell-mediated immunity as noted by a study that showed cultured DPs were present at 26% compared to 7.8% in the group without the condition [7]. Nail psoriasis is found in approximately 20% of patients with OM likely due to a disrupted nail barrier and psoriasis treatment [8-9]. Dialysis and kidney transplantation were also identified as risk factors for fungal growth due to T-cell impairment [10]. Other minor associations reported include osteoarthritis and green nail syndrome [11-12]. Various associations of OM have been elucidated in the literature, including environmental and genetic etiologies.

In addition to comorbid conditions, OM is affected by environmental factors. Those who participated in athletic activities were two-and-a-half times more likely to be diagnosed because of variables such as a moist environment, sweating, and occlusive footwear, creating ideal conditions for the proliferation of fungus [13]. Another area of interest is the effect of socioeconomic conditions and increased population density. It was determined that homeless populations had a higher incidence of non-DP OM [14]. OM also presents via many genetic influences.

One study observed a high prevalence of OM in family members with distal subungual OM in Bologna, Italy, whereas the prevalence was low among those who married into these families [15]. This finding led to investigations into the genetic basis of OM, revealing an autosomal dominant pattern of inheritance. Further studies have shown that patients with abnormal immune responses to DP often have underlying genetic defects. For example, in one study a family where four women with recurrent vulvovaginal candidiasis or OM carried the same early-stop codon mutation, Tyr238x, was identified [16]. This mutation results in a defective β-glucan receptor, dectin-1, which is crucial for recognizing fungal antigens, inducing cytokine production from monocytes/macrophages, and amplifying toll-like receptor (TLR)-2 and TLR-4 responses. Other genetic mutations observed in another study of patients with chronic OM include a reduced copy number of the defensin beta 4 gene (DEFB4), responsible for producing human beta-defensin 2 (hBD-2). Because of a -44 G/C polymorphism, it was observed by Jaradat et al. that patients had a reduced copy number of DEFB4 but elevated interleukin (IL) cd-22 and hBD-2 levels compared to controls [17]. This phenomenon can be understood as a compensatory response in patients attempting to overcome dermatophytosis rather than a direct indication of hBD-2’s efficacy in preventing it. Although IL-22 and hBD-2 are vital in the defense against fungi, a loss of copy number variations in the DEFB4 gene produces fewer variations of hBD products, leading to an inadequate response. Elevated levels of hBD-2 and IL-22 could be explained by the increased production of IL-22 as an inflammatory response to DP because IL-22 can induce hBD-2 production. In this study, it was also postulated that patients with dermatophytosis had a loss of signal transducer and activator of transcription (STAT) 3 expression, a protein responsible for inducing differentiation and increasing the sensitivity of keratinocytes to fungi. It is postulated that fewer STAT3 promoters lead to the inability to mount an adequate response against the fungi despite elevated hBD-2 and IL-22. In recent studies, the role of human leukocyte antigen (HLA) alleles’ involvement in OM is being assessed.

The HLA system, in particular HLA-DR, -DP, and -DQ, are of the major histocompatibility complex class II system and are responsible for producing antigen receptors. It detects and processes exogenous peptides, including fungal pathogens, presenting them to CD4+ T cells that mount an adaptive immune response accordingly. The arrangement of HLA alleles an individual carries determines their ability to produce an adaptive immune response to various pathogens. The lack or presence of certain HLA alleles in the individual’s genetic makeup may predispose individuals to certain conditions such as chronic OM [18]. It was observed by Asz-Sigall et al. within a Mexican mestizo population, 21 cases of OM and 41 controls, that there was a statistically significant reduction in the presence of HLA-DR6 in cases than there were in controls [19]. Although the presence of certain HLA alleles provides resistance against OM as is suggested in the study mentioned, in another study it was observed by García-Romero et al. that within another Mexican mestizo population of 47 cases and 31 healthy controls, there was a statistically significant number of cases that expressed the HLA-DR8 allele, indicating that carrying HLA-DR8 increases the risk of OM susceptibility [20]. These are a few examples of studies that show an HLA association with OM. A comprehensive meta-analysis of all the available data is needed to determine if there is a significant effect with certain HLA alleles.

In the following sections of this article, a systematic review and meta-analysis of the studies that report HLA-DR allele involvement in OM susceptibility will be provided. A combination of environmental and genetic factors plays a role in the development of OM infection. Environmental factors such as closed and moist footwear, socioeconomic conditions, slow nail growth, and contact transmission contribute to OM. A variety of genetic influences such as autosomal dominant inheritance, dectin-1 deficiency, IL-IRa deficiency, and HLA-DR profile have been implicated in OM susceptibility. Additionally, comorbid medical conditions such as type 2 diabetes mellitus, HIV infection, chronic renal failure, immunosuppression, peripheral vascular disease, and nail psoriasis contribute to creating a positive environment for OM. Although many individual studies have examined the role of HLA-DR in OM, no studies have analyzed them together to determine if there is a significant effect on OM susceptibility.

## Review

Methods

Preferred Reporting Items for Systematic Reviews and Meta-Analyses (PRISMA) guidelines were followed to create this systematic review of HLA-DR involvement in OM susceptibility. The protocol was published on August 15, 2024 [21].

Eligibility criteria

Studies that contained HLA-DR allele frequency data on patients with OM were included. Studies that contained HLA-DR alleles with too high resolution, did not provide allele frequency or odds ratio data, did not provide HLA-DR data, were written in a non-English language, or used animals as subjects were excluded. No restriction on different OM agents was placed in the inclusion of studies. No restrictions on publication date were implemented. All populations were included in this study with no restrictions placed. No restriction on different OM agents was placed in the inclusion of studies.

Information sources

Google Scholar, PubMed, and Science Direct databases were used in the literature search. All sources were last searched or consulted on February 27, 2024.

Search strategy

The following search terms were input into each of the databases: HLA OM, genetic susceptibility to OM, and HLA-DR OM. Articles were searched initially by title. If the title seemed to contain relevant information, the abstract was read. Then, if it did contain the correct information, the full article was read.

Selection and data collection process

Three reviewers independently searched the literature to find articles that met the appropriate criteria. Decisions were made as a team to determine if an article was appropriate, with the principal investigator being the final determiner in the case of a disagreement. HLA-DR allele frequency data in OM versus control cases were collected from these studies. In addition to HLA-DR allele frequency data, author name, publication year, and population ethnicity were also gathered.

Data items

Studies were determined to be included in the analysis if they compared patients with both HLA-DR and OM versus a population-matched control group containing only the HLA-DR allele. From these articles, allele frequency data, 95% confidence interval (CIs), and population characteristics such as ethnicity, study methods, fungal species, and study design were collected.

Study risk of bias assessment

Two independent reviewers assessed the risk of bias using the NIH quality assessment of case-control study tool for each source utilized and generating a table of our assessment (Table 1).

**Table 1:** National Institutes of Health quality assessment of case-control study tool

Questions	Asz-Sigall et al. [19]	García-Romero et al. [20]	Ahmed et al. [22]	Svejgaard et al. [23]	Carillo-Meléndrez et al. [24]
Was the research question or objective in this paper clearly stated and appropriate?	Yes	Yes	Yes	Yes	Yes
Was the study population clearly specified and defined?	Yes	Yes	Yes	Yes	Yes
Did the authors include a sample size justification?	No	No	No	No	No
Were controls selected or recruited from the same or similar population that gave rise to the cases (including the same time frame)?	Yes	Yes	Yes	Not indicated	Yes
Were the definitions, inclusion and exclusion criteria, algorithms, or processes used to identify or select cases and controls valid, reliable and implemented consistently across all study participants?	Yes	Yes	Yes	Yes	Yes
Were the cases clearly defined and differentiated from controls?	Yes	Yes	Yes	Yes	Yes
If less than 100% of eligible cases and/or controls were selected for the study, were the cases and/or controls randomly selected from those eligible?	N/A	N/A	N/A	N/A	N/A
Was there use of concurrent controls?	Yes	Yes	No	No	Yes
Were the investigators able to confirm that the exposure/risk occurred prior to the development of the condition or event that defined a participant as a case?	Yes	Yes	Yes	Yes	Yes
Were the measures of exposure/risk clearly defined, valid, reliable and implemented consistently (including the same time period) across all study participants?	Yes	Yes	Yes	Yes	Yes
Were the assessors of exposures/risk blinded to the case or control status of participants?	No	No	No	No	No
Were key potential cofounding variables measured and adjusted statistically in the analysis? If matching was used, did the investigators account for matching during study analysis?	Yes	No	Yes	No	No

Effect measures

HLA-DR allele frequency data were input into Review Manager version 5.4 software to generate odds ratios and forest plots with 95% confidence intervals and p-values for each HLA-DR allele. HLA-DR and subgroup analysis of each HLA-DR allele was examined.

Synthesis methods

Studies were included in the generation of summary statistics if they met the inclusion and exclusion criteria. If the studies did not contain the allele frequency data directly but provided the numbers needed to calculate them, they were calculated and included. A forest plot was generated to assess the association between various HLA genes and OM. An inverse variance statistical model and random effects analysis model were used. The data from eligible sources according to the eligibility criteria listed above were utilized. Twelve was used to evaluate the heterogeneity of the studies.

Certainty of evidence assessment

Grading of recommendations, assessment, development, and evaluations was used to determine the certainty of evidence.

Results

**Figure 1:**
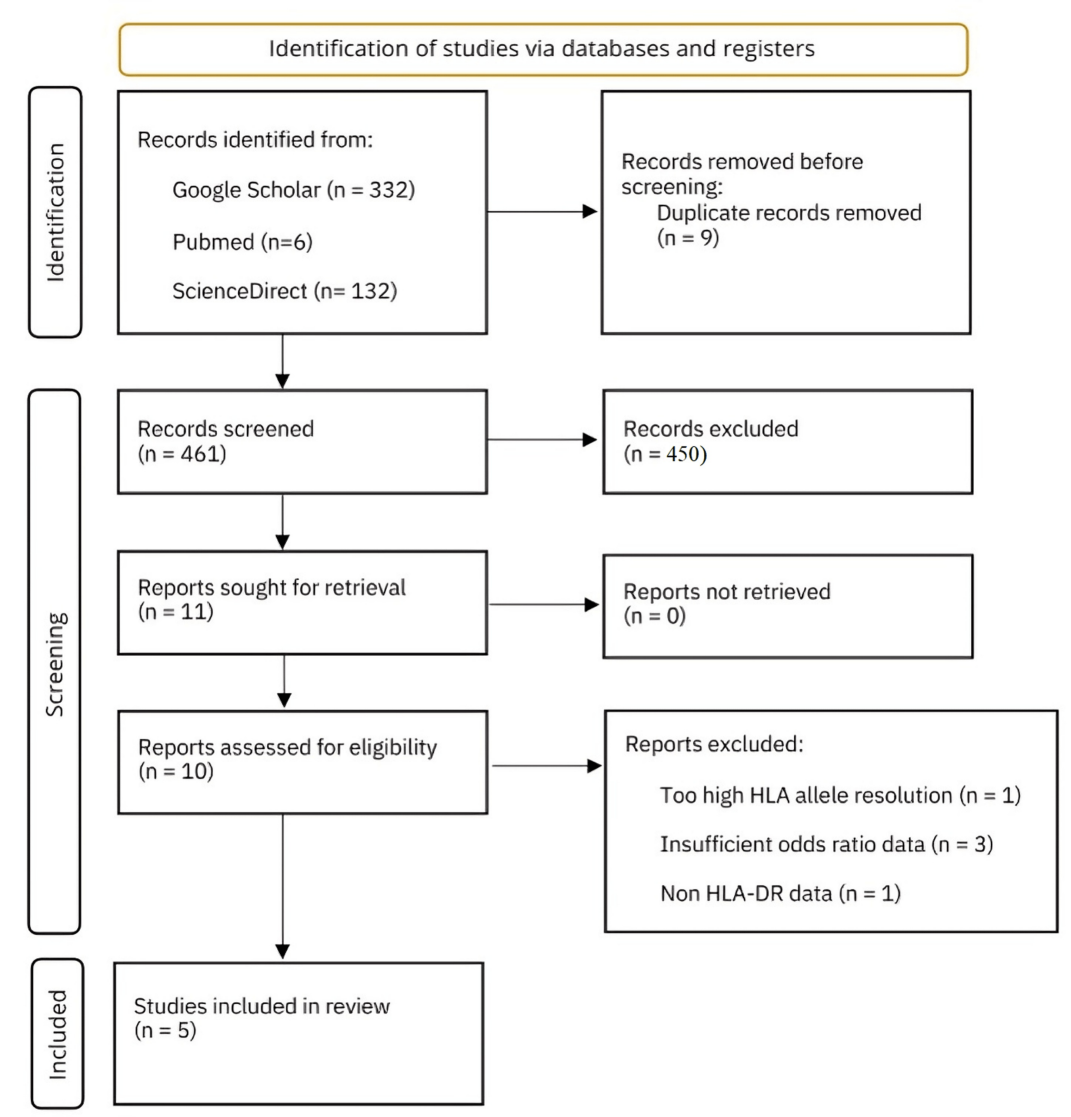
PRISMA flow diagram PRISMA: Preferred Reporting Items for Systematic Reviews and Meta-Analyses

Four hundred sixty-one records were initially screened based on titles from the Google Scholar, Scientific Direct, and PubMed databases (Figure 1). Of those, 10 articles were read and screened further to determine if they met the inclusion and exclusion criteria. Ultimately, five articles were included, with five being excluded. One article was excluded for containing too high-resolution HLA-DR allele data [25]. Three more were excluded for insufficient odds ratio data [26-28]. Finally, one more was excluded because it did not contain HLA-DR allele data [29].

Ahmed et al. aimed to investigate the relationship between immunologic factors and the persistence of chronic tinea pedis infections [22]. The study included 29 Caucasian patients with chronic tinea pedis and 558 controls matched by age and sex from the same area as the patients (Table 2). Only cases with positive microscopic visualization of hyphae on potassium hydroxide (KOH) examination and a personal or family history of atopy were included. All patients had discontinued therapy at least six weeks before the study. The controls were carefully selected to match the patient’s age, sex, and geographical location. It could not be confirmed that cases had expressed specific HLA antigens or elevated IgE levels and intercellular substance antibodies before the study because cases were included based on findings of hyphae on KOH preparations. The immunologic factors were measured using a microcytotoxicity assay. Confounding variables, including environmental factors and medications, were not included in the statistical analysis, introducing a possibility of measurement bias.

**Table 2:** HLA-DR meta-analysis study characteristics EGRET: exploration and graphics for river trends; HLA: human leukocyte antigen; KOH: potassium hydroxide; PC: personal computer; PCR: polymerase chain reaction

Author(s)	Year	Sample size	Population	Study design	Study methods	Infectious agent	HLA variants studied	Main findings
Svejgaard et al. [23]	1982	34 cases, 58-704 controls	Unspecified	Prospective case-control	Tissue typing using the 7th workshop technique for DR antigens; Fungal ID method not stated Fisher’s exact test	T. rubrum	HLA-DR 1-5, 7-10	HLA-DR does not significantly affect susceptibility to onychomycosis
Ahmed et al. [22]	1985	29 cases, 558 controls	Caucasian Mean age = 54	Prospective case-control	Cytotoxicity test; KOH Microscopy; Woolfe method (relative risk)	Unspecified	HLA-DR 1-7	In the presence of a personal or family history of atopy, HLA-DR4 demonstrates an association with onychomycosis
Asz-Sigall et al. [19]	2010	21 cases, 42 controls	Mexican-Mestizo Mean age = 40	Prospective case-control	HLA typing via PCR sequence-specific primer-based assay; Direct microscopy and culture SPSS/PC v14.0 and EGRET (odds ratio)	T. rubrum	HLA-DR 1-8	HLA-DR6 confers protection against onychomycosis
García-Romero et al. [20]	2012	47 cases, 31 controls	Mexican-Mestizo Mean age unspecified	Prospective case-control	HLA typing via PCR sequence-specific primer-based assay; Direct microscopy or culture StatCalc program in Epi (odds ratio)	Unspecified	HLA-DR 1, 3, 4, 7, 8, 10-16	HLA-DR8 increases susceptibility to onychomycosis
Carillo-Meléndrez et al. [24]	2016	25 cases, 20 controls	Mexican-Mestizo Mean age = 50	Prospective case-control	HLA typing via PCR sequence-specific primer-based assay; KOH direct microscopy and Sabouraud dextrose agar and BBL Mycosel agar culture; StatCalc program in Epi (odds ratio)	Unspecified	HLA-DR 1, 3, 4, 7, 8, 11, 13-16	Both HLA-DR8 and -DR1 are associated with increased susceptibility to onychomycosis

The possibility that chronic dermatophytosis has a genetic and immunological cause, focusing on HLA antigens, was investigated by Svejgaard et al. [23]. The study involved 34 patients with chronic dermatophytosis of the hands, feet, and nails for 3-45 years (Table 2). All had undergone long periods of medication with griseofulvin, and 26 out of the 34 patients also used ketoconazole. The control groups comprised 426-3,301 unrelated healthy individuals typed for HLA-ABC and 58-704 unrelated individuals typed for HLA-DR. It was not indicated whether the control groups or cases were matched by any factors, which introduces a selection bias (Table 1). Detailed inclusion and exclusion criteria for cases and controls were not explicitly mentioned. Tissue typing was performed using standardized NIH and seventh workshop techniques for HLA-ABC and DR antigens, respectively. It was not explicitly stated that the investigators confirmed that cases had expressed certain HLA antigens prior to the development of chronic dermatophytosis. Additionally, detailed information on whether key potential confounding variables were measured and adjusted statistically in the analysis was not provided. The primary comparison was the distribution of HLA antigens between patients and controls.

In a prospective case-control study, the HLA class II association in Mexican mestizos with *T. rubrum* OM was investigated by Asz-Sigall et al. (Table 2) [19]. The study included 21 cases and 42 controls, all recruited from the Department of Dermatology at Hospital General Dr. Manuel Gea González in Mexico City, Mexico. Participants, aged 18-60 with a mean age of 40 years, were matched by sex and age and had at least two generations of Mexican mestizo ancestry. Cases were confirmed by dermatologists and mycologists through direct microscopy and culture, and controls were selected based on a negative history of nail disease and mycology assessments to exclude subclinical OM. Both groups had no history of diabetes or immunosuppressive conditions. DNA from both groups was genotyped for eight common HLA-DR alleles using a PCR sequence-specific primer assay. Statistical analysis was performed using Fisher’s exact test and the Mann-Whitney U test, with significance set at p < 0.05. Confounding variables such as diabetes and immunosuppressive conditions were excluded from the analysis because of the selection criteria.

The aim of García-Romero et al. was to investigate the genetic factors associated with susceptibility to OM by examining the genetic polymorphism of the HLA-B and HLA-DR loci in a Mexican mestizo population and their first-degree relatives (Table 2) [20]. The study included a total of 78 subjects, with 47 having OM and a primary control group of 31 individuals who were first-degree relatives (both blood and non-blood) of the cases without OM. Additionally, a historical control group of 381 unrelated healthy individuals with 762 distinct HLA-B and HLA-DR haplotypes was used. It was noted that increasing the number of families in the study would be beneficial to avoid relying on historical controls and to include a sufficient number of healthy individuals. OM cases were confirmed through direct examination or positive DP culture. Confounding variables were addressed, both in the study’s design and through statistical methods (Table 1). Controls comprised both historical controls and first-degree relatives, which helped manage genetic background and other confounding factors. Statistical analyses, performed using the StatCalc program in the Epi Info software package (Centers for Disease Control and Prevention, Atlanta, USA), calculated odds ratios and confidence intervals to assess the association between HLA haplotypes and OM, adjusting for confounding variables. The frequency of specific HLA-B and HLA-DR haplotypes among patients and their healthy relatives was measured by extracting genomic DNA from blood samples, performing low-resolution HLA typing with sequence-specific oligonucleotide probes, and conducting statistical analyses using Epi Info.

The aim of Carillo-Meléndrez et al. was to investigate the genetic factors contributing to OM in patients with nail psoriasis (Table 2) [24]. Whereas previous research suggested that structural changes to the nail bed might explain this susceptibility, the study focused on identifying genetic predispositions. This prospective case-control study involved 25 patients and 20 controls, all selected from the Department of Dermatology at Dr. Manuel Gea González General Hospital in Mexico City. Both groups comprised Mexican mestizos, along with their parents and grandparents, ensuring a consistent genetic background for comparison. Nail psoriasis was confirmed in both patients and controls through mycological studies using KOH direct microscopy and culture, verifying OM in the patients and its absence in the controls. Although the study did not justify the sample size, the researchers acknowledged the need for a larger study to validate their findings (Table 1). HLA-DR allele presence was assessed using sequence-specific primers and polymerase chain reaction techniques.

**Figure 2:**
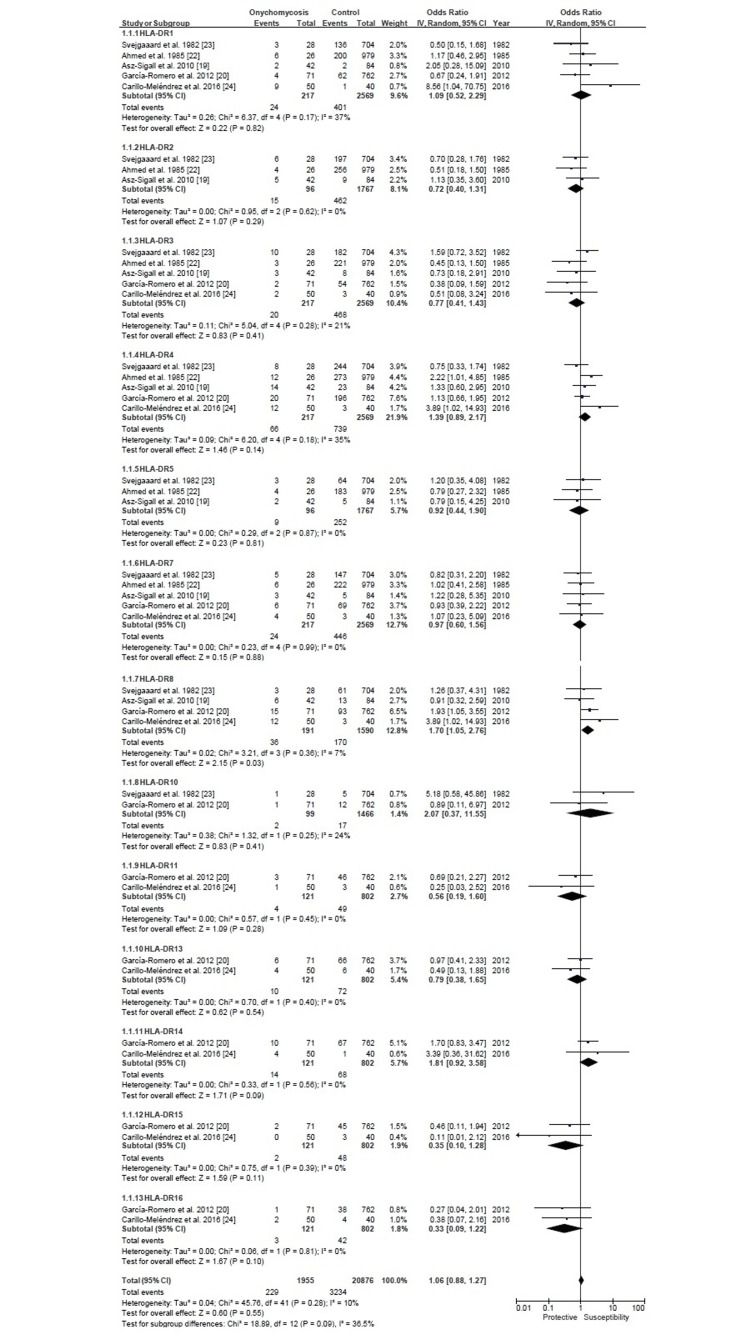
HLA-DR allele frequency forest plots HLA: Human leukocyte antigen

The forest plot analyzed the association between HLA-DR markers and the incidence of OM across five studies published between 1982 and 2016 (Figure 2). The analysis includes data on individual studies and their pooled results, providing odds ratios (ORs) and 95% CIs for the association between different HLA-DR markers and OM. The pooled odds ratios for HLA-DR markers 1-7 and 9-16 did not show a statistically significant association with OM because their confidence intervals included 1. However, HLA-DR8 showed a statistically significant association with OM, with an OR of 1.70 with a 95% CI (1.05-2.76), I2: 7%. The heterogeneity of the study results was measured through Tau², Chi², df, P, and I². These measures revealed that most subgroups of HLA-DR markers had low to moderate heterogeneity, indicating consistent results across the studies.

Discussion

OM, a fungal infection of the nail, is the most prevalent nail infection globally [4]. Genetic factors that may influence susceptibility to OM with a particular focus on HLA-DR markers have been explored in recent research. HLA-DR is a component of the major histocompatibility complex class II, a highly polymorphic genomic region that plays a crucial role in the immune system by encoding molecules involved in immune regulation and antigen presentation [30]. Because class II molecules are responsible for presenting exogenous peptides to helper CD4+ T-cells, differences in susceptibility to OM may arise if certain HLA haplotypes do not elicit an adequate T-cell response necessary for effective resistance to and resolution of the infection [20].

Numerous studies have suggested that certain HLA-DR alleles may either confer susceptibility to or provide protection against developing OM. For example, in a Mexican mestizo population, a higher frequency of HLA-DR8 among individuals with OM was found by García-Romero et al., suggesting that this haplotype may be associated with increased susceptibility to the disease [20]. Carrillo-Meléndrez et al. found that both HLA-DR8 and -DR1 significantly increased the risk of OM in patients with nail psoriasis [24]. Conversely, Asz-Sigall et al. found that HLA-DR6 was present in 33% of OM cases and 45% of controls, suggesting the presence of this allele may confer protection against infection [19].

In this systematic review and meta-analysis, the association between HLA-DR and the incidence of OM was explored. Our analysis revealed that HLA-DR1-5,7 and DR10,11,13-16 did not demonstrate a statistically significant association with OM. However, HLA-DR8 was significantly associated with the condition with an odds ratio of 1.70 (95% CI = 1.05-2.76). These findings suggest that HLA-DR8 plays a significant role in susceptibility to OM, underscoring the crucial impact of genetic factors on both the understanding and management of this condition.

These new insights may help identify at-risk populations and inform the development of future prophylactic and therapeutic interventions. Individuals identified as genetically predisposed to developing OM should be advised to avoid environmental factors such as sweating and occlusive footwear because these factors have been shown to increase risk. Furthermore, disease-associated genes may affect response to pharmacologic treatment. By tailoring therapy to an individual’s specific genetic profile, it may be possible to enhance clinical outcomes and improve the overall management of OM. For this reason, whether different HLA alleles, particularly HLA-DR8, influence responses to therapy should be studied in future research.

Although current research on HLA associations with OM, including the current study’s findings, show promise, several limitations exist. Many studies concentrate on specific populations such as the Mexican mestizo group; therefore, larger cohorts with more diverse patient populations should be studied to improve the generalizability of the findings. Another limiting factor is the heterogeneity of OM pathogenesis. For example, OM may be caused by a variety of fungal pathogens, including DP, non-DP molds, and yeasts. Consequently, the risk of developing OM may vary depending on the pathogen implicated. This may complicate the interpretation of genetic associations. Last, additional variables such as environmental factors, other genetic variations, and preexisting conditions may also influence susceptibility and potentially confound the results. Investigating gene-environment and polygenic influences would offer a more comprehensive understanding of these highly complex interactions.

In this study, the effect of the HLA-DR profile on the development of OM was evaluated. A systematic review of the literature and meta-analysis of studies containing HLA-DR allele frequency data in patients with OM was conducted. The analysis supports that HLA-DR8 was statistically significantly associated with a 70% increased risk of susceptibility to OM; OR: 1.70, 95% CI (1.05-2.76). These findings help provide a better understanding of the immunogenetic factors contributing to OM in addition to guiding targeted treatment and prevention to susceptible populations. Although this research provides an initial investigation of this topic, there are some limitations. The current data are only available in certain populations and fungal species, limiting the generalizability of the results. More research is needed to provide a better classification of the risk of developing OM in different populations.

## Conclusions

In conclusion, the current study supports a statistical basis for the HLA-DR8 allele profile being associated with OM susceptibility, providing the basis for future personalized management of this disease.
